# Decreased expression of survivin, estrogen and progesterone receptors in endometrial tissues after radiofrequency treatment of dysfunctional uterine bleeding

**DOI:** 10.1186/1477-7819-10-100

**Published:** 2012-06-01

**Authors:** Geping Yin, Tongyu Zhu, Juan Li, Ming Chen, Shujun Yang, Xiaoli Zhao

**Affiliations:** 1 Department of Obstetrics & Gynecology, Jinan Military General Hospital, #25 Shifan Road, Jinan 250031, China

**Keywords:** Dysfunctional uterine bleeding (DUB), Radiofrequency endometrial ablation (REA), Survivin, Estrogen receptor (ER), Progesterone receptor (PR)

## Abstract

****Background**:**

The purpose of the research is to study the histopathology and expression of survivin, estrogen and progesterone receptors (ER/PR) in the endometrium of patients with dysfunctional uterine bleeding (DUB) treated with radiofrequency endometrial ablation (REA).

****Methods**:**

A total of 98 DUB patients were enrolled in this case–control study. Among them, 66 underwent REA treatment and 32 optioned for hormone therapy as the control group. Immunohistochemical analysis for survivin, ER and PR expression was carried out on endometrial tissue samples collected before and 6 to 7 months after treatment for both groups.

****Results**:**

Both hormone and REA treatment ameliorated menstrual bleeding of DUB patients, with the latter showing a significantly higher effective rate. Endometrial surface tissue was replaced by fibrosis tissue in the REA treatment group. REA treatment also significantly reduced the expression of survivin, ER, and PR. Endometrial surface tissues collected from the hormone-treated control group neither showed any apparent morphological alteration nor in the expression of those receptors.

****Conclusions**:**

REA treatment changed endometrial surface tissue type from gland rich to gland poor, and significantly decreased the expression of survivin, ER, and PR. This may be an important contributing mechanism for the long-term curative effect and prevention of DUB recurrence.

## **Background**

Dysfunctional uterine bleeding (DUB) is a common gynecological disease, affecting 19% of women of child-bearing age and perimenopausal women [1,2]. The treatment options usually include drugs or surgery, both of which have significant disadvantages such as recurrence of the symptoms and dysfunctional uterus [2-5]. How to improve the efficacy of minimally-invasive surgery has become a widely-recognized concern [6,7]. Radiofrequency endometrial ablation (REA) is a minimally-invasive technique recently developed in gynecology [8-12]. After delivering a radiofrequency source into the uterine cavity a thermal effect (approximately 60-85°C) is produced which causes thermocoagulation, denaturation, and necrosis of endometrial tissue, as well as peripheral vascular thrombosis, which stops the bleeding [12].

There were histopathological studies on the treatment of menorrhagia using thermocoagulation endometrial ablation [2,6]. However, study of the histopathology and the expression for survivin, estrogen receptors (ER), and progesterone receptors (PR) in the endometrium of DUB patients after REA is lacking. Therefore, the current study was aimed to evaluate the histopathology and expression of survivin, ER, and PR in the endometrium of DUB patients, using conventional histological and immunohistochemical techniques. The association of changes of these molecules and the potential long-term effectiveness mechanism after REA treatment was assessed.

## **Method**

### **Clinical data**

#### ***Patient population***

This was a prospective study. The patient inclusion criteria were: age > 30 years without the desire of fertility, a clear diagnosis of DUB, pictorial blood loss assessment chart (PBAC) [13] >100, and benign endometrial proliferative lesions without cellular atypia confirmed by curettage pathology during the period of persistent abnormal uterine bleeding; and those who provided informed consent for treatment and research. The study was approved by the Institutional Review Board. The procedures were in accordance with the ethical standards of the responsible local or national committee on human experimentation and with the Helsinki Declaration (1975, revised 1983, World Medical Association Declaration of Helsinki). Patients who did not complete the follow-up or who had malignant gynecological cancer or other serious internal illness (including severe heart diseases, liver cirrhosis, and cerebral thrombosis) were excluded from the study. A total of 98 DUB patients were consecutively enrolled from June 2007 to June 2010. None of them had endometrial carcinoma.

The REA group consisted of 66 DUB patients, all Han Chinese, with an average age of 42.5 years (range 33–50 years), and a pre-treatment PBAC score of 182.5 ± 67.5 (time from treatment to endometrial curettage ranged from 6.5 to 12 months, with an average 9.1 ± 3.0 months). The treatment effect was defined as either a curative effect (post-treatment PBAC score <80), significant effect (PBAC score of 80–100), or treatment failure (PBAC score > 100). The rate of treatment effectiveness was defined as the sum of the curative effect and significant effect rates.

The control group was enrolled by 1:2 and consisted of 32 DUB patients who met the study eligibility criteria and opted for hormone therapy. Their average age was 43.5 years (range 32–48 years) and their pre-treatment PBAC score was 177.0 ± 67.8. Each patient began with progesterone-norethindrone (Shanghai Xinyikangjie Pharmaceutical Inc., Shanghai, China) at 5.0 mg every 8 h for 2–3 days. After the bleeding stopped, the dosage was reduced by one-third every 3 days until the daily maintenance dose of approximately 2.5-5.0 mg was reached. Patients then continued the treatment to 21 days post-hemostasis. If the symptoms of DUB recurred months later, hormone therapy was repeated. If the symptoms of DUB exacerbated, other treatment, such as hysterectomy, was considered.

Endometrial specimens were collected by curettage before treatment and post-treatment (6 to 7 months) for both the REA treated and the hormone-treated control groups.

#### ***REA treatment***

The Kangpu XVC-III gynecological RF therapy device (Xi’an Vize Electronic Technology Co. Ltd., Xi’an, China) was used for REA with the following technical parameters: 220 v ± 10%, 50 Hz ± 2%, *P* ≤ 60 w, I <2 A, 600 KHz ± 15%, power setting in the 40 w.

##### *REA procedure*

According to preoperative hysteroscopy data and guided by the color Doppler ultrasound, the coagulator was delivered to the bottom of the uterine cavity. After the coagulator was turned on, the active surface contacted the uterus membrane. From top to bottom, left to right, the coagulator was moved inside the uterine cavity in two circles, with the speed of 1 cm every 6–12 s. When moved to the left or right, the distance was the width of a coagulator. Movement was made between the anterior and posterior wall of the uterus three times, and the left and right side wall once (the whole procedure generally took 10 to 15 min to complete).

### **Histopathology and immunohistochemical assay**

Reagents and materials include: (1) conventional histological reagents including paraffin embedding materials, H&E staining solutions, and so on; and (2) for immunohistochemistry, mouse monoclonal antibodies against human survivin, ER, and PR (NeoMarkers Products, USA) and universal streptomycin avidin-peroxidase hypersensitive (SP) staining kit (Santa Cruz Products, Germany).

#### ***Histology and immunostaining***

Endometrial biopsy samples were embedded with paraffin, and serial sections at 4 μm thickness were prepared. Some sections underwent regular H&E staining. Other sections were processed for immunostaining for survivin, ER, and PR, as described in details below.

Sections were deparaffinized, rehydrated, and underwent antigen retrieval process using ethylenediaminetetraacetic acid (EDTA) solution. After washing in 0.1 M PBS, sections were incubated overnight in primary antibodies for survivin, ER or PR at 4°C. The sections were then incubated successively in biotinylated secondary antibody (reagent C) for 10 min; streptomycin-peroxidase solution (reagent D) for 10 min; and followed by freshly prepared DAB/H2O2 solution from the staining kit for 2–10 min. After a further wash in PBS, the sections were counterstained with hematoxylin, dehydrated, and mounted with neutral gum for microscopic examination and photography. Methodological controls included using known positive sections as the positive control, and omission of primary antibodies or their replacement with PBS as the negative control.

#### ***Assessment of immunohistochemical assay***

(1) Survivin immunohistochemical staining scoring criteria (ISSC): Survivin expression was primarily localized in the cytoplasm of endometrial epithelial cells and occasionally in the nucleus. Brown-yellow granules in the cytoplasm and/or nuclei indicated positive (positive cells). Ten randomly selected fields were examined under light microscope for each section. The scoring was based on the following two scales: A. The intensity of staining: 0 points for no staining, 1 point for weak staining, 2 points for moderate staining, and 3 points for intense staining; B: The percentage of positive cells in all cells counted: 0 point if positive cell is ≤ 5%, 1 point = 6-25%, 2 points = 26-50%, and 3 points if ≥ 51%. The combination of these two scales for each section was used as the score for survivin immunostaining (ranged between 0–9 in total). This score was considered as the level of survivin expression in the study, and the mean and standard deviation were calculated for each group.

(2) ER and PR ISSC: ER and PR expression were mainly localized in the nucleus of endometrial epithelial cells and stromal cells. Brown-yellow granules in the nucleus and/or cytoplasm indicated positive (positive cells). Ten randomly chosen fields were examined under light microscope for each section. The percentage of positive cells was quantified by counting 100 cells in total for each field. The mean and standard deviation of the percentage of positive cells were calculated for all fields examined.

### **Statistics**

The Student *t*-test was used for continuous data, and the *χ*^2^ test was used for categorical data. Commercially available software was used to perform the tests (SPSS 13.0). Statistical significance was defined as *P* <0.05.

## **Results**

### **The effect on DUB of the hormone-treated control and the REA-treated groups**

There was no statistical difference between REA and the control group on geographic distribution, ethnicity, age, pre-treatment PBAC scores, and time from treatment to curettage (*P* > 0.05). In the control group, the PBAC score decreased from 177.0 ± 67.8 at baseline to 120 ± 70.2 at 8.5 months (8.5 ± 2.5 months) post-treatment, which was statistically significant (*P* < 0.05). The total effective rate of DUB was 56.3% (18/32 cases- there were 14 patients whose symptoms of DUB recurred months later, exacerbated after another course of hormone therapy and eventually undertook hysterectomy). In the REA group, the PBAC score decreased from 182.5 ± 67.5 at baseline to 56.7 ±22.0 at 9.1 months (9.1 ± 3.0 months) post-treatment, which showed a greater significant difference (*P* < 0.01). In addition, the reduction of the PBAC scores from baseline to post treatment follow-up was significantly higher in the REA group (125.8 ± 44.5) than in control group (57.2 ± 37.5) (*P* < 0.01). Furthermore, the total effective rate of the REA group reached 95.5% (63/66 cases), which was also higher than that in the control group (95.5% *vs.* 56.3%, *P* < 0.05). These results indicate that REA had better treatment effects than the hormone therapy.

### **Endometrial histopathology before and 6 to 7 months after REA**

**Figure 1:**
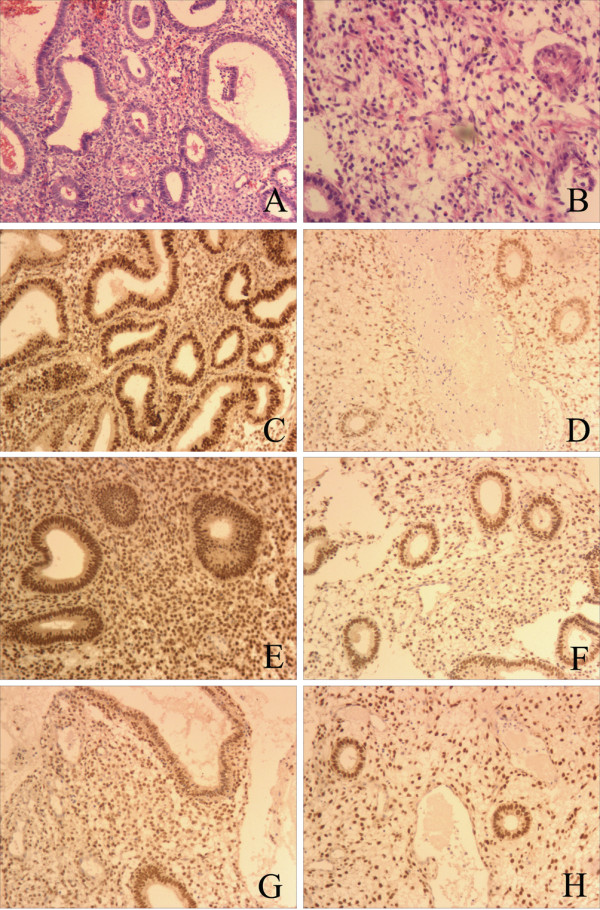
**Endometrial pathology and survivin, ER, and PR expression before and 6 to 7 months after REA treatment in DUB patients with complex endometrial hyperplasia. **(**A**) Before REA, the endometrium showed complex hyperplasia, ‘back to back’ glandular hyperplasia, leather bag shape glandular enlargement. (**B**) Six to seven months after REA, glandular tissue became fibrous tissue with scarce glands and blood vessels in endometrial curettage. (**C**) Before REA, the expression of survivin (strong positive). (**D**) Six months after REA, the expression of surviving showed weak positive. (**E**) Before REA, the expression of ER (strong positive). (**F**) Six months after REA, the expression of ER showed weak positive. (**G**) Before REA, the expression of PR (strong positive). (**H**) Six months after REA, the expression of PR showed weak positive. DUB, dysfunctional uterine bleeding; ER, Estrogen receptors; PR, Progesterone receptor; REA, Radiofrequency endometrial ablation.

Tables 1 and 2 list changes of the pathology type before and after treatment in both groups. Prior to treatment, DUB patients in the REA group showed benign proliferative endometrial glands, some of which were irregularly enlarged and closely arranged into a ‘back to back’ shape (Figure 1A). After 6 to 7 months of REA, most endometrial tissues were shown to be replaced by granulation tissue, and only small amounts of glands remained which were small in size, and surrounded by fibrosis tissue (Figure 1B).

**Table 1:** The changes of the pathology type before and after treatment in control groups

**Histopathological types**	**Control groups *n *(%)**		**Statistics**	
	**Before treatment**	**After treatment**	***χ***^**2**^	***P***
Fibrous connective tissue and granulation tissue	0 (0.0%)	0 (0.0%)	-	-
Some endometrial glands and granulation tissue	0 (0.0%)	0 (0.0%)	-	-
Proliferative endometrium	8 (25.0%)	15 (46.9%)	3.326	>0.05
Simple hyperplasia	14 (43.8%)	12 (37.5%)	0.259	>0.05
Complex hyperplasia	10 (31.2%)	5 (15.6%)	2.177	>0.05

Statistics is Fisher probabilistic method (compared with before treatment). *n*, number of cases.

**Table 2:** The changes of the pathology type before and after treatment in REA groups

**Histopathological types**	**REA group *n *(%)**		**Statistics**	
	**Before treatment**	**After treatment**	***χ***^**2**^	***P***
Fibrous connective tissue and granulation tissue	0 (0.0%)	20 (30.3%)	23.030	<0.01
Some endometrial glands and granulation tissue	0 (0.0%)	27 (40.9%)	32.832	<0.01
Proliferative endometrium	16 (24.2%)	13 (19.7%)	0.461	>0.05
Simple hyperplasia	30 (45.5%	4 (6.1%)	25.611	<0.01
Complex hyperplasia	20 (30.3%)	2 (3.0%)	17.220	<0.01

Statistics is Fisher probabilistic method (compared with before treatment).

*n*, number of cases; REA, radiofrequency endometrial ablation.

*χ*^2^ test showed that there was no significant difference in the endometrial pathological types between the two groups before any treatment (*P* > 0.05). In the control group there was no significant difference in the pathological types before and after the hormone treatment (*P* > 0.05). In contrast, there was a significant difference between the pathological types before and after treatment in the REA group (*P* < 0.01). In addition, REA treated group showed a significant difference in the pathological types when compared with the control group (*P* < 0.01).

### **Expression of survivin, ER, and PR expression before and 6 to 7 months after treatment in the control group**

The analysis showed that prior to the hormone treatment, the average expression for all three molecules (survivin, ER, and PR) were higher in endometrial tissues with complex hyperplasia than those with simple hyperplasia or proliferative endometrium, but the differences were not significant (*P* > 0.05). The mean levels of three molecules slightly decreased after the hormone treatment, but again the differences were not significant (*P* > 0.05) (Table 3).

**Table 3:** Endometrial tissues survivin, ER, and PR expression before and after treatment in the control group

**Pathology before treatment**	***n***	**Before treatment (mean ± SD)**			**After treatment (mean ± SD)**		
		**Survivin scores**	**ER (%)**	**PR (%)**	**Survivin scores**	**ER (%)**	**PR (%)**
Proliferative endometrium	8	5.5 ± 1.2	53.3 ± 18.1	40.6 ± 27.3	5.7 ± 1.8	55.6 ± 30.1	51.8 ± 37.0
Simple hyperplasia	14	6.1 ± 1.8	58.1 ± 16.3	44.7 ± 262	5.9 ± 1.6	57.6 ± 34.3	50.7 ± 30.1
Complex hyperplasia	10	8.5 ± 2.0	86.2 ± 36.2	85.3 ± 21.5	6.7 ± 2.3	82.7 ± 32.1	77.1 ± 36.4

The comparisons were between before and after treatment.

*n*, number of cases; ER, estrogen receptor; PR, progesterone receptor.

### **Expression of survivin, ER, and PR before and 6 to 7 months after treatment in REA treatment group**

Before REA, the expression of survivin was strong in the cytoplasm of glandular cells (Figure 1C). After REA (6 to 7 months follow-up), it was weak (Figure 1D) (Figure 1 E, F, G, H, Table 4).

**Table 4:** Endometrial tissues survivin, ER, and PR expression before and after treatment in the REA group

**Pathology before treatment**	***n***	**Before treatment (mean ± SD)**			**After treatment (mean ± SD)**		
		**Survivin scores**	**ER (%)**	**PR (%)**	**Survivin scores**	**ER (%)**	**PR (%)**
Proliferative endometrium	16	5.7 ± 1.7	53.3 ± 18.3	45.9 ± 22.3	2.5 ± 1.7^a^	23.8 ± 10.4^a^	11.3 ± 7.5^a^
Simple hyperplasia	30	7.2 ± 1.7	51.4 ± 15.7	54.2 ± 25.7	1.8 ± 0.9^a^	18.9 ± 14.4^a^	26.9 ± 11.3^a^
Complex hyperplasia	20	8.1 ± 2.3	79.9 ± 20.4	86.7 ± 31.6	2.9 ± 0.3^a^	18.3 ± 12.1^a^	21.4 ± 6.8^a^

The comparisons were between before and after treatment.

^a^*P* <0.05.

*n*, number of cases; ER, estrogen receptor; PR, progesterone receptor; REA, radiofrequency endometrial ablation.

Before treatment, the expression levels of survivin, ER, and PR were not significantly different between the REA and control groups (*P* > 0.05). After treatment, there was a significant reduction in the expression of these three molecules in the REA group (*P* < 0.05). The differences in the levels of survivin, ER, and PR expression before and after treatment were also greater in the REA group than those in the control group (*P* < 0.05).

### **Complications associated with RF procedure and treatment/prevention measures**

Abdominal pain occurred within 24 h after treatment in 30.3% (20/66) of RF subjects. Endometrial coagulation necrosis can stimulate uterine cramps and contractions that may result in abdominal pain, usually occurring within 12 h of treatment. Patients were treated for abdominal pain with atropine (1.0 mg intramuscular injection).

The incidence of post-treatment intrauterine adhesions was 7.6% (5/66); no severe adhesions presented. At 1 and 3 months post-operation, the uterine cavity was examined for adhesions using a No. 4 cervical dilator. In young women, an intrauterine device (IUD) was inserted as a means to separate the adhesions, or the adhesions were separated under hysteroscopy. Infection control was provided with the prescription of routine antibiotics for 1 to 2 weeks after treatment; no severe endometritis or pelvic inflammatory disease occurred among the patients.

## **Discussion**

The endometrium of patients with anovulatory DUB is continuously stimulated by estrogen without the antagonism of progesterone, resulting in various degrees of proliferative changes followed by repeated bleeding [1]. Thermalcoagulation effects of REA can directly inactivate the estrogen and progesterone receptors in the lesion. However, the literature on the expression of estrogen and progesterone receptors and survivin in the repaired tissues at 6 to7 months after treatment is lacking. This might be important in understanding whether REA can provide long-term effects beyond temporary hemostasis.

### **The action of REA treatment on DUB**

In medical physics, sinusoidal alternating current with a frequency greater than 100 kHz is called radio frequency (RF). As RF can create a thermal effect (approximately 60-85°C) in biological tissues at an approximate depth of treatment of 5–7 mm, a coagulator can cause the following changes in endometrial tissue to achieve the purpose of DUB treatment: (1) direct thermal coagulation and necrosis or apoptosis of the endometrial functional layer and the basal cell layer; (2) peripheral intravascular coagulation around the lesion and bleeding cessation; and (3) gradual replacement of endometrial tissues by fibrous connective tissue that do not ablate periodically. The histopathology data from this study showed decreases in simple hyperplasia and complex hyperplasia, and increases in gland-free fibrous connective tissue and granulation tissue with fewer endometrial glands in the endometrium following REA treatment.

### **The effects of REA on survivin, ER, and PR and potential mechanism for long-term effectiveness on DUB**

The survivin gene is the only one in the inhibitor of apoptosis proteins (IAP) family that was found to be related to both apoptosis and the regulation of the cell cycle. Studies have shown that survivin has partial or complete low expression in the normal endometrium, which may be due to the active proliferation of endometrial cells [14,15]. The expression of PR and ER in endometrial cells of patients with anovulatory DUB was found higher than normal, and was positively correlated with the degree of endometrial proliferation [16,17].

The histopathologic results of the study showed that REA can directly cause the coagulation, necrosis, collapse, occlusion, and disappearance of glandular lumen in the endometrium. The endometrium was gradually replaced by fibrous granulation tissue that does not ablate periodically, which may lead to the reduction of menstrual flow or amenorrhea. Immunohistochemical data of the study showed that the expression levels of survivin, ER, and PR in fibrous granulation tissue after REA treatment were significantly reduced, and were also significantly lower than those in the hormone treated control group. At 6 to 7 months post-treatment, the expression of survivin, ER, and PR from the scattered glands in the fibrous endometrium remained low, which is likely to result in a diminished response to estrogen and progesterone stimulation. The decreased expression of the receptors would suggest a suppressed binding ability to estrogen and progesterone. Whether the suggestion is another mechanism for the DUB recurrence-prevention and long-term effectiveness of REA treatment needs further studies.

## **Conclusions**

REA treatment changed endometrial surface tissue type from gland rich to gland poor, and significantly decreased the expression of survivin, ER, and PR. This may be an important contributing mechanism for the long-term curative effect and prevention of DUB recurrence.

## **Abbreviations**

DUB: Dysfunctional uterine bleeding; EDTA: Ethylenediaminetetraacetic acid; ER: Estrogen; HE: Hematoxylin-eosin; ISSC: Survivin immunohistochemical staining scoring criteria; PBAC: Pictorial blood loss assessment chart; PR: Progesterone receptors; REA: Treated with radiofrequency endometrial ablation.

## **Competing interests**

The authors declare that the authors have no competing interests.

## **Authors contributions**

GY conceived and designed the experiments, performed the experiments and wrote the paper. JL and SY performed the experiments. TZ analyzed the data. MC and XZ contributed materials and analysis tools. All authors read and approved the final manuscript.

## References

[B1] Amir E Richard HS Dysfunctional uterine bleeding 2010 http://emedicine.medscape.com/article/795587-overview

[B2] Apgar BS Kaufman AH George-Nwogu U Kittendorf A Treatment of menorrhagia Am Fam Physician 2007 75 1813 1819 17619523

[B3] Hodgson DA Feldberg IB Sharp N Cronin N Evans M Hirschowitz L Microwave endometrial ablation: development, clinical trials and outcomes at three years Br J Obstet Gynecol 1999 106 684 694 10.1111/j.1471-0528.1999.tb08368.x 10428525

[B4] Li D The curative effect of RU486 for the treatment of dysfunctional uterine bleeding (in Chinese - English abstract available) Chin J Mod Drug Appl 2009 3 104 105

[B5] Xiu-min F Clinical analysis of mifepristone treatment of 68 cases of perimenopausal dysfunctional uterine bleeding (in Chinese - English abstract available) Medical Information 2010 23 905 906

[B6] Zarek S Sharp HT Global endometrial ablation devices Clin Obstet Gynecol 2008 51 167 175 10.1097/GRF.0b013e3181621f97 18303511

[B7] Taskin O Onoglu A Inal M Turan E Sadik S Vardar E Postaci H Wheeler JM Long-term histopathologic and morphologic changes after thermal endometrial ablation J Am Assoc Gynecol Laparosc 2002 9 186 190 10.1016/S1074-3804(05)60130-2 11960046

[B8] Thijssen RF Radiofrequency induced endometrial ablation: an update Br J Obstet Gynaecol 1997 104 608 613 10.1111/j.1471-0528.1997.tb11541.x 9166206

[B9] Dequesne JH Gallinat A Garza-Leal JG Sutton CJ van der Pas HF Wamsteker K Chandler JG Thermoregulated radiofrequency endometrial ablation Int J Fertil Womens Med 1997 42 311 318 9406837

[B10] Clark TJ Samuel N Malick S Middleton LJ Daniels J Gupta JK Bipolar radiofrequency compared with thermal balloon endometrial ablation in the office: a randomized controlled trial Obstet Gynecol 2011 117 109 118 10.1097/AOG.0b013e3182020401 21173651

[B11] El-Nashar SA Hopkins MR Creedon DJ Cliby WA Famuyide AO Efficacy of bipolar radiofrequency endometrial ablation vs thermal balloon ablation for management of menorrhagia: A population-based cohort J Minim Invasive Gynecol 2009 16 692 699 10.1016/j.jmig.2009.06.022 19896595 PMC3770134

[B12] Yin GP Chen M Shao X Zhu TY Wang YZ Radiofrequency minimally invasive treatment of uterine benign diseases (in Chinese - English abstract available) Progress Obstetrics and Gynecology 2003 12 200 203

[B13] Higham JM O’Brien PM Shaw RW Assessment of menstrual blood loss using a pictorial chart BJOG 1990 97 734 739 10.1111/j.1471-0528.1990.tb16249.x 2400752

[B14] Takai N Miyazaki T Nishida M Nasu K Miyakawa I Survivin expression correlates with clinical stage, histological grade, invasive behavior and survival rate in endometrial carcinoma Cancer Lett 2002 184 105 116 10.1016/S0304-3835(02)00190-8 12104054

[B15] Chen X Zhang Z Feng Y Fadare O Wang J Ai Z Jin H Gu C Zheng W Aberrant survivin expression in endometrial hyperplasia: another mechanism of progestin resistance Modern Pathology 2009 22 699 708 10.1038/modpathol.2009.25 19287462

[B16] Hu K Zhong G He F Expression of estrogen receptors ERalpha and ERbeta in endometrial hyperplasia and adenocarcinoma Int J Gynecol Cancer 2005 15 537 541 10.1111/j.1525-1438.2005.15321.x 15882182

[B17] Kalogiannidis I Bobos M Papanikolaou A Makedos A Amplianitis I Vergote I Nenopoulou E Makedos G Immunohistochemical bcl-2 expression, p53 overexpression, PR and ER status in endometrial carcinoma and survival outcomes Eur J Gynaecol Oncol 2008 29 19 25 18386458

